# Metabarcoding reveals distinct microbiotypes in the giant clam *Tridacna maxima*

**DOI:** 10.1186/s40168-020-00835-8

**Published:** 2020-04-21

**Authors:** Isis Guibert, Gael Lecellier, Gergely Torda, Xavier Pochon, Véronique Berteaux-Lecellier

**Affiliations:** 1 Swire Institute of Marine Science, The University of Hong Kong, Hong Kong, SAR China; 2 UMR250/9220 ENTROPIE IRD-CNRS-UR, Promenade Roger-Laroque, Sorbonne Université, Noumea Cedex, New Caledonia France; 3 USR3278 PSL CRIOBE CNRS-EPHE-UPVD, Papetoai, Moorea, French Polynesia; 4 UVSQ, Université de Paris-Saclay, 45 Avenue des Etats-Unis, Versailles Cedex, France; 5 ARC, Centre of Excellence for Coral Reef Studies, James Cook University, QLD, Townsville, 4811 Australia; 6 Coastal and Freshwater Group, Cawthron Institute, Private Bag 2, Nelson, 7042 New Zealand; 7 Institute of Marine Science, University of Auckland, Private Bag 349, Warkworth, 0941 New Zealand

**Keywords:** Giant clams, Assemblages, Microbiome, Symbiodiniaceae, Microbiotype

## Abstract

**Background:**

Giant clams and scleractinian (reef-building) corals are keystone species of coral reef ecosystems. The basis of their ecological success is a complex and fine-tuned symbiotic relationship with microbes. While the effect of environmental change on the composition of the coral microbiome has been heavily studied, we know very little about the composition and sensitivity of the microbiome associated with clams. Here, we explore the influence of increasing temperature on the microbial community (bacteria and dinoflagellates from the family Symbiodiniaceae) harbored by giant clams, maintained either in isolation or exposed to other reef species. We created artificial benthic assemblages using two coral species (*Pocillopora damicornis* and *Acropora cytherea*) and one giant clam species (*Tridacna maxima*) and studied the microbial community in the latter using metagenomics.

**Results:**

Our results led to three major conclusions. First, the health status of giant clams depended on the composition of the benthic species assemblages. Second, we discovered distinct microbiotypes in the studied *T. maxima* population, one of which was disproportionately dominated by Vibrionaceae and directly linked to clam mortality. Third, neither the increase in water temperature nor the composition of the benthic assemblage had a significant effect on the composition of the Symbiodiniaceae and bacterial communities of *T. maxima*.

**Conclusions:**

Altogether, our results suggest that at least three microbiotypes naturally exist in the studied clam populations, regardless of water temperature. These microbiotypes plausibly provide similar functions to the clam host via alternate molecular pathways as well as microbiotype-specific functions. This redundancy in functions among microbiotypes together with their specificities provides hope that giant clam populations can tolerate some levels of environmental variation such as increased temperature. Importantly, the composition of the benthic assemblage could make clams susceptible to infections by Vibrionaceae, especially when water temperature increases.

Video abstract.

## Background

Giant clams (*Hippopus* and *Tridacna* genera) are emblematic and keystone species of Indo-West Pacific coral reef ecosystems. These filter-feeding organisms play a wide range of ecological roles: their calcium carbonate shell is a substrate for colonization, they provide food for numerous reef organisms, act as a shelter, and contribute to primary production on the reef [1, 2]. Like some other marine bivalves, giant clams live in close partnership with unicellular dinoflagellate algae from the family Symbiodiniaceae [3, 4] that satisfy the majority of the clams’ carbon and energy needs [5, 6]. This partnership with Symbiodiniaceae is established horizontally (acquired from the environment), only after metamorphosis from larva to juvenile [7]. Formerly known as nine clades of a single dinoflagellate genus (*Symbiodinium* [8]), seven clades have recently been re-classified to the genus level [9]. Microbiome profiling studies using the ITS2 and/or the LSU nuclear and chloroplast markers have recorded clade A (genus *Symbiodinium*), C (genus *Cladocopium*), D (genus *Durusdinium*), and G (genus *Gerakladium*) in giant clams [10–12]. These genera are also found in cnidarians [13, 14], but in contrast to the typically intracellular symbiosis with corals, algae reside in the clams’ siphonal mantle extracellularly [7].

Giant clams can harbor one single algal genus or an assemblage of multiple genera [10, 15]. While *Tridacna crocea* is predominantly associated with one algal genus at a time (*Symbiodinium*, *Cladocopium*, or, less frequently, *Durusdinium*), *Tridacna squamosa* and *Tridacna maxima* typically harbor multiple genera simultaneously [10, 11], except in the Red Sea where they exclusively associate with *Symbiodinium* spp. [16]. This species-specific symbiosis with Symbiodiniaceae can be disrupted by environmental change that—similar to corals—can lead the expulsion or apoptosis of the photosynthetic symbionts [17–20] and cause clam bleaching and, subsequently, death. Indeed, mass bleaching and mortality of giant clams related to thermal stress and high solar irradiance, often associated with extremely low tides, have been recorded in the past [21, 22]. Bleaching has been widely studied in Scleractinia, and it has been shown that the composition of the Symbiodiniaceae community in corals shifts in response to environmental changes [23–26]. However, this is a complex system, and data on the stable partnership between adult corals and newly acquired Symbiodiniaceae are still lacking [27, 28]. Contrary to corals, however, only few studies have scrutinized the nature of the symbiosis between Symbionidaceae and tridacnids. DeBoer and collaborators [10] showed that giant clams that harbor *Symbiodinium* (formerly known as “clade A”), a typical temperature- and light-resistant algal genus in corals, are more sensitive to thermal and light stress than those that harbor *Cladocopium*. This result is, however, inconsistent with a recent report on tridacnids of the Red Sea, where *Symbiodinium* was found as the unique algal genus in clams that lived in high temperature and salinity conditions [16]. The role and potential flexibility of the Symbiodiniaceae assemblage of giant clams need clarification in order to better understand the threats and adaptive capacity of these important reef organisms.

It is increasingly recognized that symbiotic microorganisms other than Symbiodiniaceae also greatly contribute to the physiological performance of complex marine metaorganisms, such as the coral holobiont [29], or, plausibly, giant clams. The prokaryotic microbial community, for example, plays a significant role in the coral’s nutrient cycling and immune defense (reviewed in [30, 31]). Similarly to the Symbiodiniaceae community, the composition of the prokaryotic microbial community can change with the coral’s environment [32–36] even though this is not supported by all studies [37, 38]. Bacterial community changes are not always beneficial; however, opportunistic pathogenic taxa, such as Vibrionaceae, can colonize corals and lead to coral disease and the death of the colony [35, 39, 40]. While the abundance of metagenomic studies on corals describes the diversity of microbes in the coral holobiont, their exact roles and functions remain unclear [41, 42]. Even less is known about the prokaryotic community of bivalves, where most microbial studies have focused on pathogenic bacteria that cause disease or mortality [43–45] or pose a human health risk via direct consumption [43, 46, 47]. Bivalves filter through large volumes of water for feeding and hence accumulate a diverse suite of microorganisms that are not directly associated with their normal physiology, making it particularly challenging to understand the composition and role of the clam core microbiome. Only one study has recently reported the bacterial composition of different tissues of Tridacninae [48]. Assuming a similarly important role of microbes in the healthy functioning and adaptive capacity of the bivalve holobiont as recognized in corals, it is critical to better understand what influences the composition of the clam microbiome.

While the initial establishment of the Symbiodiniaceae community occurs horizontally in the early stages of the giant clam’s life [7, 49], nothing is known about its bacterial community. In corals, it is well established that a part of its bacterial microbiome is vertically transmitted (passed on from parent to the offspring, e.g., [50, 51]) and part of it is acquired horizontally, and therefore highly depends on the environment (e.g., [32, 52]). The Symbiodiniaceae composition of the reef benthos has been found to influence the Symbiodiniaceae composition of some symbiotic metazoans, e.g., nudibranchs [53]. Furthermore, the associated bacteria and Archaea of coral species have also been shown to vary according to the presence of certain marine organisms (e.g., macroalgae [54]) or their absence (e.g., decrease of coral cover and overfishing [55]). Thus, the environmental microbiome (dinoflagellate algae as well as prokaryotes) that is available for uptake might also greatly depend on the surrounding existing benthic communities. To test the importance of the benthic assemblage composition on the makeup of the giant clam’s microbiome as well as their physiological performance, we created artificial coral reef assemblages comprising of the clam *Tridacna maxima* and two common Indo-Pacific scleractinian coral species, *Pocillopora damicornis* and *Acropora cytherea*. The fitness of *T. maxima* was monitored in different assemblages under control and increased water temperature conditions, and its associated bacterial and algal communities were characterized using 16S, ITS2, and 23S profiling.

## Results

### Mortality of giant clams

**Fig. 1:**
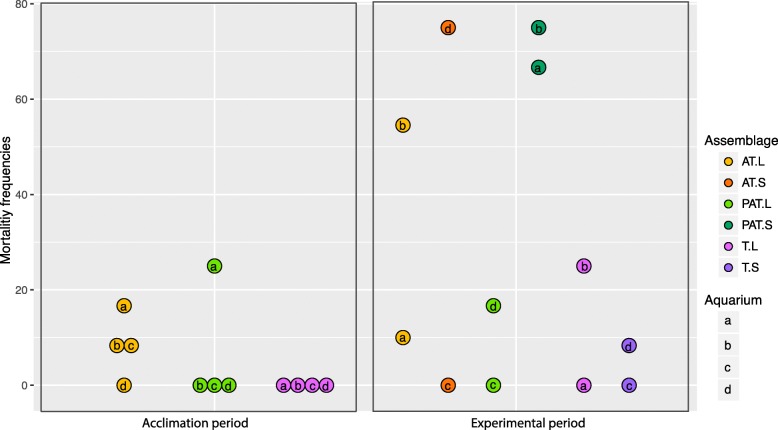
Mortality rates of giant clams in three experimental benthic assemblages during the acclimation period and during the experimental period. Each circle represents one aquarium. PAT, *P. damicornis*, *A. cytherea*, and *T. maxima*; AT, *A. cytherea* and *T. maxima*; PT, *T. maxima*; T, *P. damicornis* and *T. maxima*; L, lagoon temperature; S, thermal stress; a–c, aquarium for each assemblage and conditions

Some mortality of *T. maxima* was observed during the experiments (Fig. 1; Additional file 1). During the 12 days of the acclimation period, no mortality was observed in T assemblages (four aquaria), and some mortality of clams was observed in three of four aquaria with AT assemblages and one of four aquaria with PAT assemblages. A logit analysis did not reveal significant differences in mortality rate among assemblages (*p* = 0.9263) with a range of response (death probability) from 8.3 10^–2^ to 6.3 10^–2^ and 5.4 10^–10^ for AT, PAT, and T. During the 5 days following acclimation, clam mortality was observed with frequencies varying according to thermal conditions (L or S) and assemblage composition (*p* = 0.003). Mortality was highest for AT (L and S) and PAT (S) assemblages with a range of response of 0.33, 0.39, and 0.71.

### The bacterial community of giant clams

The microbiome of 36 giant clams was characterized. Due to mortality, sample numbers differ among experimental conditions (number of analyzed giant clams: *n* = 8 in T at the end of acclimation (T0) and *n* = 3 in AT.L, *n* = 3 in AT.S, *n* = 6 in PAT.L, *n* = 4 in PAT.S, *n* = 6 in T.L, and *n* = 6 in T.S at day 17 (T1)). Seven individuals showed signs of declining health (decrease in closure reactivity, loss of color, and mantle degradation) and therefore were further labeled as “dying.”

**Fig. 2:**
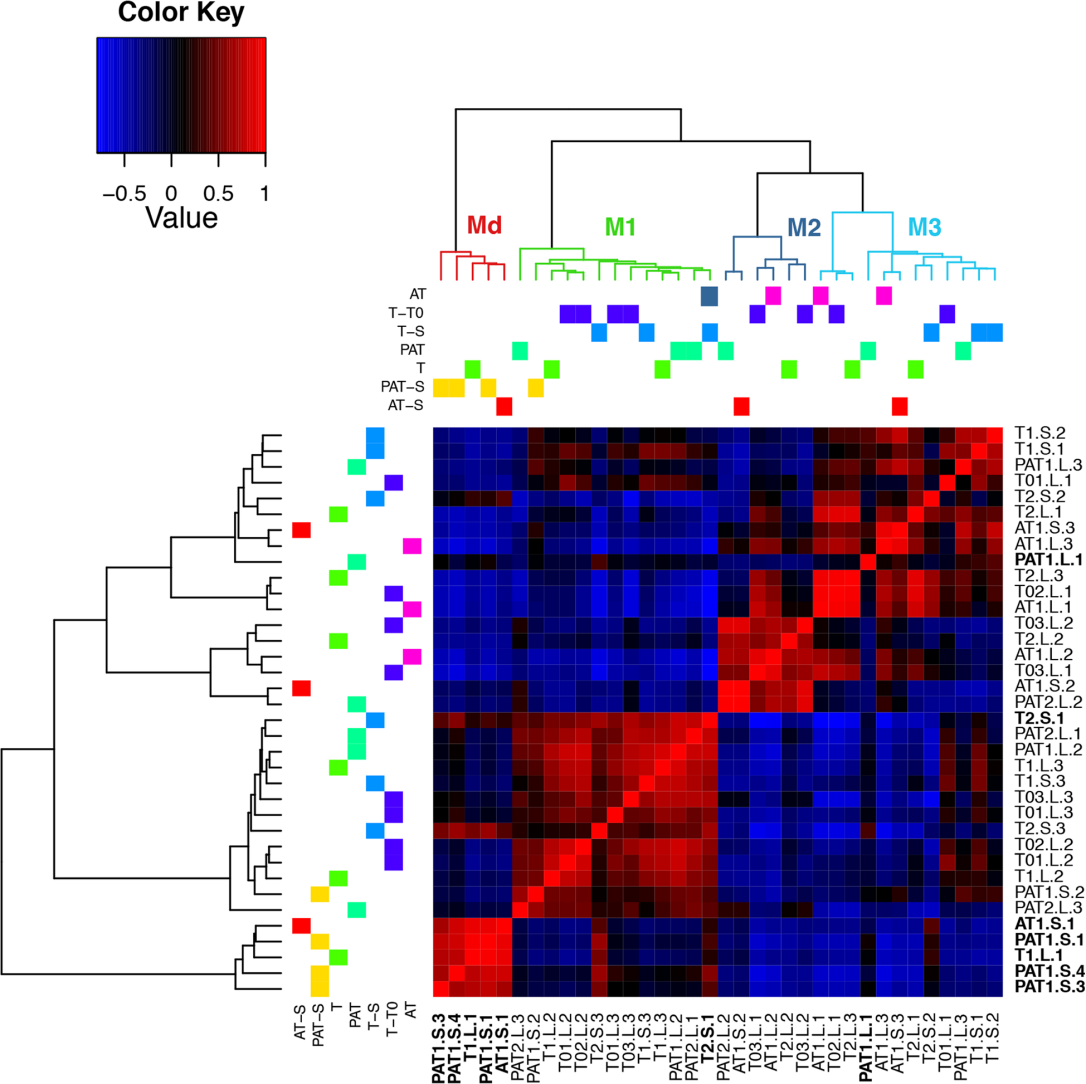
Heatmap from the pairwise correlation between microbiomes of clams in experimental benthic communities and at control and elevated temperature. PAT, *P. damicornis*, *A. cytherea*, and *T. maxima*; AT, *A. cytherea* and *T. maxima*; T, *T. maxima*; 0, control; (1–3), experiment number; L, lagoon temperature; S, thermal stress; (1–4), sample number. M, microbial community clusters (microbiotypes); Md, dying clam microbiotype; 1–3, clam microbiotype number. Dying clam names are in bold

The 16S DNA gene libraries yielded 716,100 sequences from which 693,609 sequences (8911–33,115 per sample) were selected with an average length of 448 bp. The selected sequences were assigned to 43 phyla subdivided in 415 bacterium families (Additional file 2). The family-level pairwise correlation of microbiomes among samples yielded four distinct microbial community clusters, hereafter referred to as microbiotypes (Fig. 2). The microbiotypes did not correlate with neither the composition of the experimental assemblages (chi-squared test, Monte Carlo *p* = 0.14) nor the thermal conditions (*p* = 0.12). However, one microbiotype (Md) was exclusively characteristic of dying clams (five of seven dying clams shared this microbiotype). Two further dying clams did not cluster under Md, but instead fell into the microbiotype M1 (T2.S.1) and M3 (PAT1.L.1), while also sharing similarities with Md, as well as two other healthy clams from the S treatment (T2.S.2; T2.S.3). Most of the diversity has been covered in every cluster even though the representation of the microbial community is not fully complete, for some samples (Additional file 3). The number of detected species was significantly positively correlated with the number of reads in the sample (Pearson correlation test, 0.35; *p* = 0.03).

Bacterial species richness measured by chao1 (Additional file 4) ranged from 1205 to 5934 per sample, with the lowest average bacterial species richness found in M1 (1682 ± 366). The average index of bacterial species richness was slightly higher in Md (2386 ± 1922) and increased greatly in M3 and M2 (3057 ± 1344 and 3294 ± 1363, respectively). Thus, significant differences were determined between M1 and M2–M3 (pM1:M2 = 0.0072; pM1:M3 = 0.0056). Species diversity, measured by the Inverse Simpson Index (Additional file 4), ranged from six to nine in microbiotypes M1, M3, and Md and was significantly different from M2 (56.5; pM1:Md = 0.0018; pM1:M2 = 0.0013; pM1:M3 = 0.0005).

**Fig. 3:**
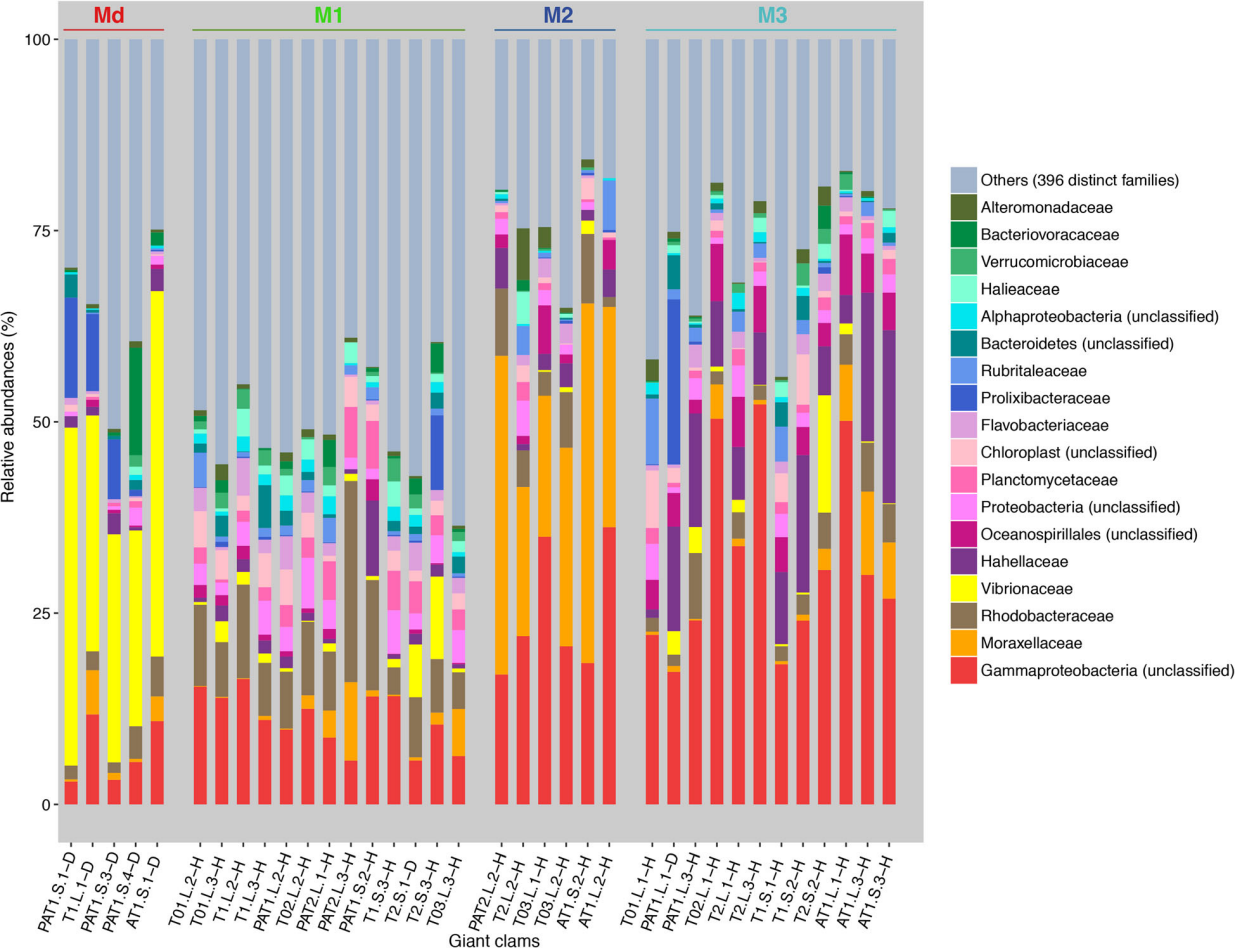
Relative abundance of the 18 most abundant bacterial families in giant clams *T. maxima* grouped by microbiotype; 396 less abundant taxa are grouped under “others.” Abbreviations of experimental assemblages: PAT, *P. damicornis*, *A. cytherea*, and *T. maxima*; AT, *A. cytherea* and *T. maxima*; T, *T. maxima*; 0, control; (1–3), experiment number; L, lagoon temperature; S, thermal stress; (1–4), sample number; D, dying clam; H, healthy clam; M, microbiotypes; Md, dying clam microbiotype; 1–3, clam microbiotype number

The most abundant bacterial family in the Md microbiotype was Vibrionaceae (Fig. 3; > 35% on average), and within that, *Catenococcus* spp. were present in four samples (T1.L1, 89.9%; PAT1.S.1, 83.2%; PAT1.S.4, 67%; and PAT1.S.3, 58.5%). The M1 microbiotype was characterized by a higher proportion of Rhodobacteraceae (57.4%) and a lower proportion of Gammaproteobacteria (20.4%). The M2 microbiotype was dominated by Moraxellaceae (71.2%) and an unclassified Gammaproteobacteria (21.1%). The M3 microbiotype harbored a higher level of Gammaproteobacteria (53.7%) combined with a higher abundance of Hahellaceae (73%).

### Bacterial functional roles

**Fig. 4:**
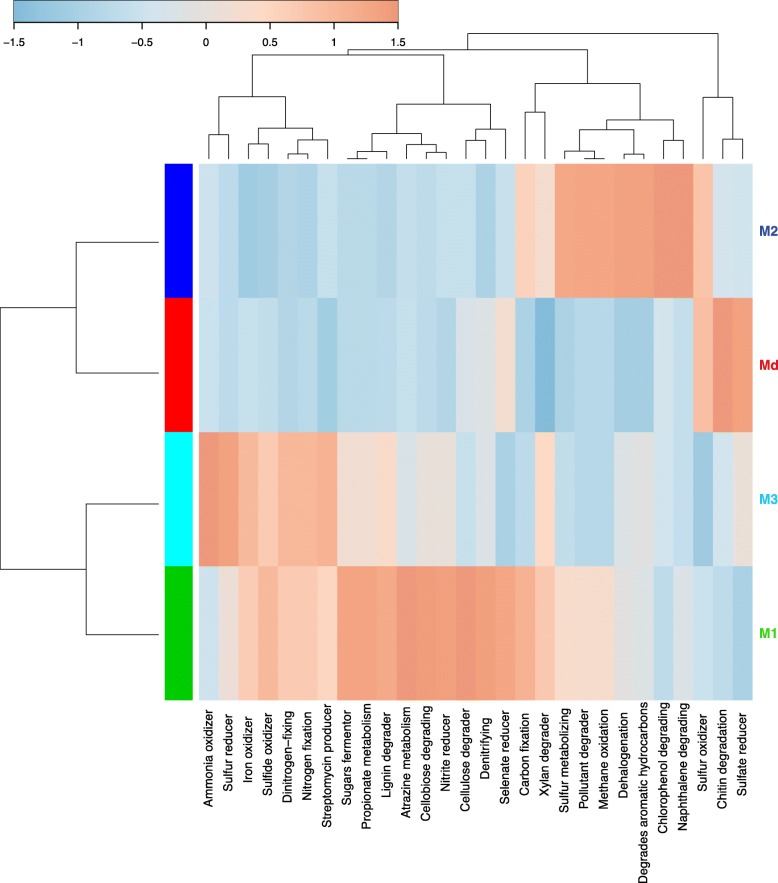
Taxonomy-based functional profiling of bacterial communities in giant clams (*Tridacna maxima)* by microbiotype. Enrichment (red) and decrease (blue) of functions are presented on a relative scale using a Pearson distance measure and the average clustering method. M, microbiotypes; Md, dying clam microbiotype; 1–3, clam microbiotype number

To scrutinize putative functional differences of bacterial communities of *T. maxima* in experimental treatments, we applied a taxonomy-based profiling using METAGENassist (Fig. 4) and PICRUSt2 (Additional file 5). This functional clustering clearly distinguished Md, M1, M2, and M3 microbiotype according to the relative proportion of functions. Md was mainly characterized by activities responsible for the degradation of organic material, particularly an increase in sulfate and selenate reducers and chitin degradation. Sulfur oxidizer was a shared function with M2. The microbiotype M2 was characterized by enhanced sulfur and methane metabolism, dehalogenation, and functions related to the degradation of various aromatic molecules (e.g., naphthalene and chlorophenol). The M1 and M3 microbiotypes both had an increase in processes involved in sulfur and nitrogen metabolism, and lignin and xylan degradation. The diversity of enhanced functions involved in nitrogen metabolism was higher in M3, while those involved in saccharide and polyphenol metabolism were higher in M1.

### The Symbiodiniaceae composition of giant clams

The composition of the Symbiodiniaceae community was determined for 36 giant clam samples by Illumina sequencing of the ITS2 DNA region (49,318 ± 17,083 reads/sample, 1,775,437 sequence reads in total) and the 23S rRNA gene (86,541 ± 31,373 reads/sample, 3,115,502 sequences in total). After filtering, 1,209,269 ITS2 sequences were kept with a median length of 289 bp, representing 45,951 unique sequences, while 2,740,830 23S gene sequences were kept with a median length of 196 bp representing 29,420 unique sequences. The average number of ITS2 and 23S sequences per sample was 33,591 and 76,134, respectively.

The ITS2 marker identified *Symbiodinium*, *Cladocopium*, and *Durusdinium*, while the 23S marker detected *Symbiodinium*, *Cladocopium*, and *Gerakladium* in our giant clam samples (Additional file 6). With both markers, *Symbiodinium* was present in all 36 samples and was the dominant clade in all except two samples, where *Cladocopium* was the dominant algal genus. At a higher taxonomic resolution, we found two species of *Symbiodinium* with ITS2, A6, and A1, with A1 only found as the background species. Only one species in each of *Cladocopium* (C66) and *Durusdinium* (D17) were assigned in our samples. The 23S marker discriminated only one species in each of the algal genera present: A3, C3, and G9. *Symbiodinium* A6 (ITS2) and A3 (23S) were found in all samples. *Cladocopium* C3 (23S) co-occurred with *Cladocopium* C66 (ITS2) except for seven of 23 samples where only C66 was detected at low levels. *Durusdinium* D17 and *Gerakladium* G9 were detected in only 3 samples, AT1.L.1, AT1.S.1, and AT1.S.3, and T03.L.2, AT1.L.1, and AT1.S.3, respectively. Overall, Symbiodiniaceae composition did not correlate to any of the four microbiotypes or any experimental condition.

## Discussion

Microbiomes, including viruses, bacteria, and fungi, are an integral part of multicellular organisms, contributing to their health and physiological performance. Despite a surge of interest in this research focus, very few invertebrate microbiomes have been studied, with the notable exception of insects. Among marine organisms, marine bivalves, especially oysters because of their economic value, are part of the few marine invertebrates of which microbial community has been studied [56–59]. In this study, we tested the influence of different benthic species assemblages on the microbial community and health of giant clams under two environmental contexts, by the concomitant analysis of the clams’ bacterial and algal microbiome. Hence, the current study has characterized for the first time the bacterial microbiome of *T. maxima*, from French Polynesia. The microbiome of giant clams is particularly interesting because clams are exposed to an extreme abundance and diversity of microbes through filter feeding, and because they live in symbiosis with dinoflagellate algae. We showed that the presence of the coral *Acropora cytherea* in an assemblage negatively affects the health of the giant clam *Tridacna maxima* and that this effect is amplified under temperature stress. Our results showed that nearly all clams with compromised health were characterized by a distinct microbiome in which the Vibrionaceae family was enriched. Interestingly, the bacterial community of healthy clams fell in three clusters irrespective of the composition of the benthic assemblage, the clams’ symbiotic algal composition, or water temperature. Our discovery of specific microbiome structure, detectable from the genus level, is the first description of microbiotypes in invertebrates.

### The composition of benthic species assemblages influences the health of giant clams

The first remarkable result of this work is that the frequency of clam mortality, associated with a *Vibrio* infection, is correlated with the benthic species that surrounded them. When *A. cytherea* was present in the assemblage (PAT and AT), clam mortality increased. This pattern was particularly striking for PAT under increased temperature. Since all aquariums were filled with seawater from the same pipe and some healthy giant clams in T assemblages harbored the Vibrionaceae species at a lower proportion, the prevalent hypothesis is an increased susceptibility of infection due to the presence of corals. Benthic species, particularly corals, are highly competitive [60] and have been classified based on their aggressiveness [61–64]. Corals compete either by direct physical contact or via the production and secretion of secondary metabolites that can weaken or kill neighboring organisms [65–70]. These metabolites are produced by the coral host itself or by their associated microorganisms, some of which are known to synthetize toxic compounds [67, 71]. Other than the direct effect of a toxic metabolite potentially produced by *A. cytherea* or by its associated organisms, the decline and subsequent death of giant clams could also be the consequence of anti-inflammatory molecules produced by corals [72, 73], reducing the immune response of clams against microbial pathogens. An immune depression associated with the reproductive period and/or increase of water temperature has also been linked to vibriosis in poikilotherm organisms, including mollusks [74–77]. Few marine studies have recorded *Catenococcus* spp. [78–80]. However, this member of the Vibrionaceae family is described as a pathogen in the seaweed *Kappaphycus alvarezii* in which infection by *Catenococcus thiocyli* causes bleaching [79]. Bacterial extracts from sponge *Stylotella* sp. of Proteobacteria closely related to *Catenococcus thiocycli*, showed toxicity activity [80]. As nearly all dying clams harbored a typical microbiome mainly composed of Vibrionaceae (*Catenococcus* spp. or an unclassified genus), we hypothesize that the combined effect of secondary metabolites from *Acropora* corals and increased water temperature may have weakened the clams’ defenses against *Vibrio* infection. Interestingly, the microbiome of two healthy clams, from T assemblage under thermal stress, showed similarities to that of dying clams. The relative proportion of Vibrionaceae in these two clams is higher than in other members of their microbiotype (11–15% of total bacterial families, compared with < 3% in other healthy clams and 25–50% in the majority of dying clams), suggesting that these clams might have had a compromised health without visible symptoms yet. Our results suggest that the relative proportion of Vibrionaceae could be used as an early indicator of clam health in natural populations.

### Microbiotypes in giant clams

We used multiple genetic markers for profiling the Symbiodiniaceae diversity in giant clams, the advantages of which have recently been highlighted by Pochon et al. [12]. As expected, using both *ITS2* and *23S* markers, we found a higher level of taxonomic diversity, including the detection of free-living Symbiodiniaceae species in clam samples. Indeed, beyond the identification of *Symbiodinium* and *Cladocopium* species, *ITS2* and *23S* allowed for the detection of *Durusdinium* and *Gerakladium* species in some of our samples. In accordance with Pochon et al. [12], *Symbiodinium tridacnidorum* ([81]; former subclade A6/A3) was the dominant species in all but two of 36 samples. In these remaining two samples, subclade C66/C3 of *Cladocopium* species was dominant. Interestingly, *Durusdinium* was detected in our young giant clam samples while not detected before in adult clams from French Polynesia supporting the hypothesis that this algal genus is restricted to juvenile clams [11, 82]. Importantly, the composition of the clam Symbiodiniaceae community did not show any correlation with the composition of the experimental benthic assemblages, nor to thermal condition or to a particular microbiotype. Similarly, microbiome analysis did not highlight any link with species assemblages or thermal status. The major bacterial phyla found in the microbiome of *T. maxima* are those commonly found in marine invertebrates. Among them are Proteobacteria that assist in food digestion in bivalves and contribute to the host’s nitrogen uptake [83] and Bacteroidetes that play an important role in bivalve immunity by limiting the establishment of potentially pathogenic bacterial species [84]. The most abundant class was Gammaproteobacteria, and the most abundant families were Moraxellaceae and Rhodobacteraceae. Representatives of these families are present in the marine environment and some of them are described as symbionts of aquatic organisms such as certain bacteria of the Rhodobacteraceae family [59]. Interestingly, similarly to human enterotypes [85], *T. maxima* individuals harbored distinct microbiotypes (M1–M3). All three microbiotypes found in this study were clearly defined by their bacterial community composition. This variability in bacterial community composition could result from multiple inter-organismal interactions such as host-specific immune capacity allowing the presence or absence of bacteria incompatible with the presence of other bacterial genera [86–89]. These differences in microbial community composition, together with the systematic co-occurrence of certain bacterial taxa, create distinct functional biomarkers to the clam host. Thus, microbiotypes can use different pathways to achieve similar overall functions. For example, sulfur metabolism mostly involved sulfur reduction and sulfide oxidizing in M3, sulfide oxidizing in M1, and sulfur oxidizing, as well as an undetailed sulfur metabolic pathway in M2. Some of the most enriched functions were also specific to a given microbiotype, thus providing to its host-metabolic capacities. For example, M1 and M3 were mostly characterized by lignin and xylan degradation with an emphasis on cellulose degradation in M1. The most characteristic functions in M2 are related to pollutant degradation (aromatic compounds such as naphthalene or chlorophenol). We found all three microbiotypes in every experimental benthic assemblage, under both control and increased temperature, and in samples issued from the two different cohorts, suggesting that similarly to humans, microbiotypes are not driven by the environment or genetic factors. In fact, we found that at least three microbiotypes could exist in two distinct cohorts of clams collected in the same lagoon. This suggests that different microbiotypes can most likely confer similar functions to host organisms that allow them to thrive in the same environment. Metabolic pathways linked to microbiotypes presumably confer some specific functional properties to their clam host, and therefore will likely influence the adaptive capacity of clam populations to environmental change. Importantly, even though there was no change in the bacterial community during our thermal stress experiment, our results do not rule out that a more intense thermal stress would influence the composition of the *Tridacna maxima* microbiome, similarly to sponges (e.g., [90, 91]) and oysters [92].

Our work suggests that the diversity of species assemblages and thus the composition of the coral reef benthos, together with increasing water temperatures, could negatively impact the health of giant clams and potentially of other reef organisms. Our findings therefore support the idea that, similarly to terrestrial conservation and restoration, preserving entire benthic assemblages should be the goal of marine conservation strategies.

## Materials and methods

### Sample collection

Four colonies of each of the two coral species, *A. cytherea* and *P. damicornis*, were collected in the Moorea lagoon, French Polynesia (Linareva site, 17° 30′ S, 149° 50′ W [93]), and fragmented in 45 nubbins each. As described in Guibert et al. [94], two cohorts of the giant clam *T. maxima* (cohort 1: 4-cm-long individuals, *n* = 150; cohort 2: 8-cm-long individuals, *n* = 70) were bought from a clam nursery (N° Tahiti: 139 519) on Reao Island (18° 28′ S, 136° 25′ W). The coral nubbins and giant clams were kept on underwater racks for 8 months in the Moorea lagoon, at the coral nursery ground of the InterContinental Moorea Resort & Spa. Each species was kept on a separate rack at 3 m depth below chart datum; the racks were separated by 3 m to minimize interspecific interactions during this acclimation period.

**Fig. 5:**
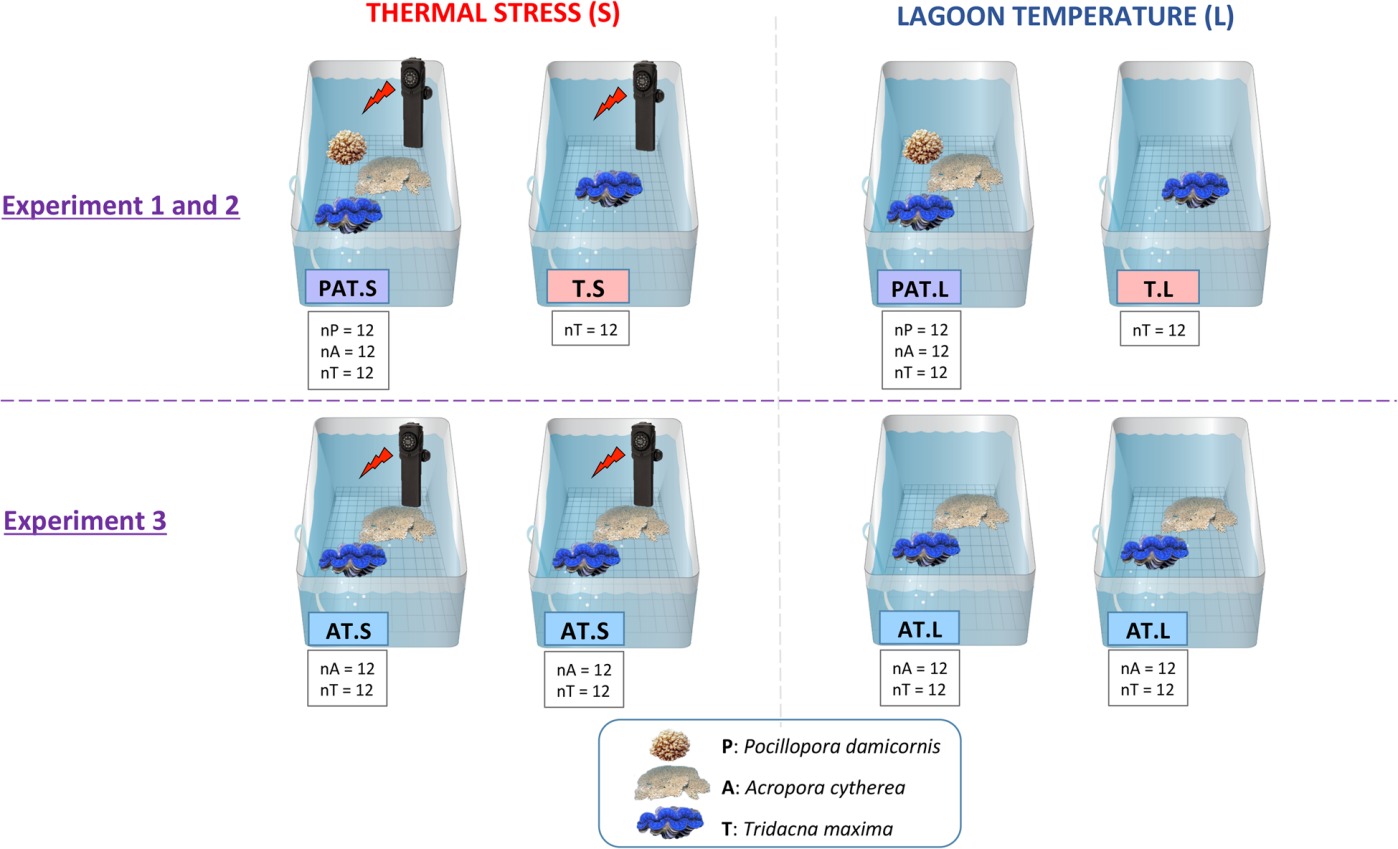
Experimental design of the thermal stress experiments (1, 2, and 3) composed of either one, two, or three species: *P. damicornis* + *A. cytherea* + *T. maxima* (PAT); *A. cytherea* + *T. maxima* (AT); and *T. maxima* alone (T). N, number of organisms

We constructed artificial benthic assemblages for thermal stress experiments composed of either one, two, or three species: *P. damicornis* + *A. cytherea* + *T. maxima* (PAT); *A. cytherea* + *T. maxima* (AT) and *T. maxima* alone (T) (Fig. 5). To set up the experiments, 12 specimens of each species of a given assemblage were used: three nubbins from each of four distinct colonies per coral species and 12 giant clam individuals. In addition, 3 additional clams were placed in T assemblages for each experiment (*n* = 9). Two open-circuit 40-L aquaria were set up per assemblage, with seawater pumped directly from Moorea’s Opunohu Bay at a flow rate of 20 L/h and filtered through two successive filters of 60 μm to remove sediments. After 12 days of acclimation (T0) at lagoon temperature (approximately 27 °C, L), one aquarium per assemblage was maintained at lagoon temperature while thermal stress (S) was applied to the second one. The temperature was increased by 1 °C per day until reaching 32 °C at day 17 (T1). The water temperature was controlled in each aquarium with the Biotherm pro system (Hobby, Stukenbrock, Germany). Temperature data were recorded every 10 min with temperature/light data loggers (P/N U22-001, Onset, Bourne, MA, or Ruskin, Ottawa, Canada). All aquaria received the same light using LED lamps (PlanetPro ELOS, Verona, Italy), following a diurnal cycle.

The 9 additional giant clams were sampled at day 12 (T0), and 28 individuals were sampled randomly at day 17 (T1). A small piece of the mantle was sampled and stored in 70 % ethanol at − 20 °C until molecular analysis. The remaining parts of the clams’ mantle were stored at − 80 °C for further studies. The experiments were performed twice for each benthic species assemblage, spaced 2 weeks apart, except for the AT assemblage which was also studied in duplicate but during the same experiment. For each of the experiments, the aquaria were randomly chosen to avoid a potential aquarium effect. The health of giant clams was monitored daily by visual observations (closure reactivity, color, mantle aspect), and mortalities were recorded.

### DNA extraction, PCR amplification, and sequencing

Genomic DNA of giant clams from the main study were isolated following the bench protocol for animal tissues (DNEasy Blood and Tissue Kit, Qiagen) after rinsing the sample with sterile freshwater. Three different genetic markers were selected for metabarcoding. The bacterial 16S ribosomal RNA gene’s V3/V4 region was amplified with the “Bakt_341” forward (5′-CCTACGGGNGGCWGCAG-3′) and “Bakt_805” reverse (5′-GACTACHVGGGTATCTAATCC-3′) primers [95]. Symbiodiniaceae diversity was analyzed with two genetic markers. The internal transcribed spacer 2 (ITS2) region of the nuclear ribosomal array was amplified with the “itsD” forward (5′-GTGAATTGCAGAACTCCGTG-3′) and “ITS2-Rev2” reverse (5′-GCCTCCGCTTACTTATATGCT-3′) primers [96]. The chloroplast cp23S-rDNA domain V region was amplified with the forward primer “23SHYPERUP” (5′-TCAGTACAAATAATATGCTG-3′) and the reverse primer “23SHYPERDN” (5′-GTTATCGCCCCAATTAAACAGT-3′) [97]. The primers were modified to include Illumina™ overhang adaptors as described by Kozich et al. [98].

Polymerase chain reactions were performed using 10 to 50 ng of template DNA in 50 μL volumes with the reaction mixture containing 5 μL GC enhancer, 1 μL of each primer (10 pmol/μL), and 30 μL AmpliTaq Gold® 360 PCR Master Mix (Life Technologies, Carlsbad, CA). After a denaturing step of 3 min at 95 °C, 40 or 45 cycles consisting of 30 s at 94 °C, 30 s at 52 °C, and 90 s at 72 °C were performed followed by a final elongation at 72 °C for 7 min. Amplicons were visualized on a 2% agarose gel. Samples were purified using magnetic beads (Agencourt Bioscience Corporation, Grand Rapids, Michigan), quantified (Qubit® 2.0 Fluorometer, Invitrogen, Carlsbad, CA), and diluted to 3 ng/μL concentration. Internal quality controls were used with two amplicons from DNA samples of *Ciona savignyi* and *Asterias amurensis* [99]. Nuclease-free UltraPure™ water (Thermo Fisher Scientific, Waltham, MA) was used as a negative control. The final HTS library preparation and paired-end run sequencing (MiSeq Illumina™ platform, 2 × 250 bp) were performed by New Zealand Genomics Ltd. at the University of Auckland. Sequencing failed for one sample (T02.L3; 781 reads), which was removed from the analysis.

Raw data are available on BioProject, no. PRJNA494911 (NCBI).

### Microbial community analysis

Bacterial community analyses were performed using the MiSeq standard operating procedure (SOP) in MOTHUR (v1.39.5; http://www.mothur.org/) to produce operational taxonomic units (OTUs) [98]. Briefly, sequence reads were assembled into contigs and amplicon adaptors removed, then trimmed to improve quality (quality control ≥ 35) and split into two groups based on genetic markers (bacterial 16S and 23S). Unique sequences were selected and counted for bacterial operational taxonomic units (OTUs). Sequences were aligned to the SILVA full-length sequences and taxonomic references (Silva-vr128) using the *K*-mer search method [100]. The “screen.seqs” command was used to remove insertions or deletions, and the sequences were filtered to remove the overhangs at both ends. Unique sequences were selected again to eliminate the potential redundancy created during sequence trimming. A pre-cluster step (2-bp difference) was performed, and the VSEARCH algorithm was employed to remove chimeras [101]. The SILVA database was used to assign sequences with a cutoff level of 80, and mitochondria and chloroplast assignments were removed. Operational taxonomic units were then clustered under a 0.03 cutoff level and phylotypes were determined by taxonomic classification. Rarefaction curves were calculated using rarecurve function in the vegan R package v2.6-13 with a subsampling of 20 and 100 permutations.

To analyze the alpha diversity, samples were subsampled to 8911 sequences and then clustered into OTUs with a cutoff of ≤ 0.03. Chao1 and Inverse Simpson Indices were performed in MOTHUR [102–104]. To assess the putative functional profiles of the bacterial community of giant clams, METAGENassist [105] was used for automated taxon-to-phenotype mapping. During METAGENassist’s own data processing, data filtering was based on interquartile range [106] and variables with over 50% zeroes were removed. The 288 remaining variables (OTUs) were normalized over sample by sum and range scaling. Data were analyzed for “metabolism by phenotype” using a Pearson distance measure and an average clustering method to visualize the top 28 features in a heatmap.

The bacterial community analysis was also preformed according to a second approach: the gene content inference with a single-nucleotide resolution by using the dada2 version 1.12.1 [107] followed by the functional prediction with PICRUSt2 [108] from the dada2 ASV table. The scripts are described in Additional file 7.

Analysis of the Symbiodiniaceae community was adapted from Arif et al. [109]. Sequences were annotated via BLASTN using an ITS2 database [109] and a custom 23S database (Additional file 8) [110].

### Statistical analysis

All statistical analyses were performed using R v3.4.2 [111, 112]. For the bacterial correlation analysis, the raw counts per sample were homogenized to counts per million and centered. Pairwise correlation was performed with the Pearson method and clustered with the Ward method. The relative abundances of bacterial families were performed following Röthig et al. [113] and using Venny for determining the presence/absence of bacterial families (2.1.0, http://bioinfogp.cnb.csic.es/tools/venny/index.html). Statistical analysis of PICRUSt2 functional profiles was performed with STAMP [114]. The Symbiodiniaceae community composition was analyzed following the same workflow as for bacterial relative abundances. Data manipulation and visualization was done using the R packages (reshape2 and ggplot2). A logistic regression (logit) was performed on the giant clams’ health data.

## Supplementary information

**Additional file 1.** Numbering of remaining giant clams in the aquariums during the acclimation period and experimental period according to health status. d0: seeding in the aquarium, Alive: number of healthy giant clams, Death= number of dying giant clams, Pred: death by predation. PAT: *P. damicornis*, *A. cytherea* and *T. maxima*; AT: *A. cytherea* and *T. maxima*; T: *T. maxima*; L: lagoon temperature; S: thermal stress.

**Additional file 2.** Metabarcoding results of bacteria. PAT: *P. damicornis*, *A. cytherea* and *T. maxima*; AT: *A. cytherea* and *T. maxima*; T: *T. maxima*; 0: control; (1-3): experiment number; L: lagoon temperature; S: thermal stress; (1-4): sample number.

**Additional file 3.** Rarefaction curves of sequences from the different microbiotypes (Md, M1, M2 and M3).

**Additional file 4.** Summary statistics of 16s DNA-based bacterial community composition of giant clams according to their groups. Based on subsampled sequences (n=8,411). G: groups; AVG: average; SD: standard deviation; PAT: *P. damicornis*, *A. cytherea* and *T. maxima*; AT: *A. cytherea* and *T. maxima*; T: *T. maxima*; 0: control; (1-3): experiment number; L: lagoon temperature; S: thermal stress; (1-4): sample number.

**Additional file 5.** Principal Component Analysis build from PICRUSt2 analysis on the bacterial communities of *T. maxima* according to the microbiotypes. M: microbiotypes, Md: dying clam microbiotype; 1-3: clam microbiotype number.

**Additional file 6.** Relative abundance of Symbiodiniaceae subclades in *Tridacna maxima* determined by Illumina sequencing of the ITS2 DNA region (a) and the 23S rRNA gene (b). Abbreviations of experimental assemblages: PAT: *P. damicornis*, *A. cytherea* and *T. maxima*; AT: *A. cytherea* and *T. maxima*; T: *T. maxima*; 0: control; (1-3): experiment number; L: lagoon temperature; S: thermal stress; (1-4): sample number.

**Additional file 7.** Script of the bacterial community analysis performed with dada2.

**Additional file 8.** 23s custom database.

## Data Availability

Supplemental information for this article can be found at Microbiome Journal. All data are available upon request. Raw data are available on BioProject, no. PRJNA494911 (NCBI)
