# Supplementary figures and images for: Metabarcoding reveals distinct microbiotypes in the giant clam Tridacna maxima
Source: Microbiome. 2020 Apr 21;8:57. doi: 10.1186/s40168-020-00835-8 (PMC7175534; doi:10.1186/s40168-020-00835-8)

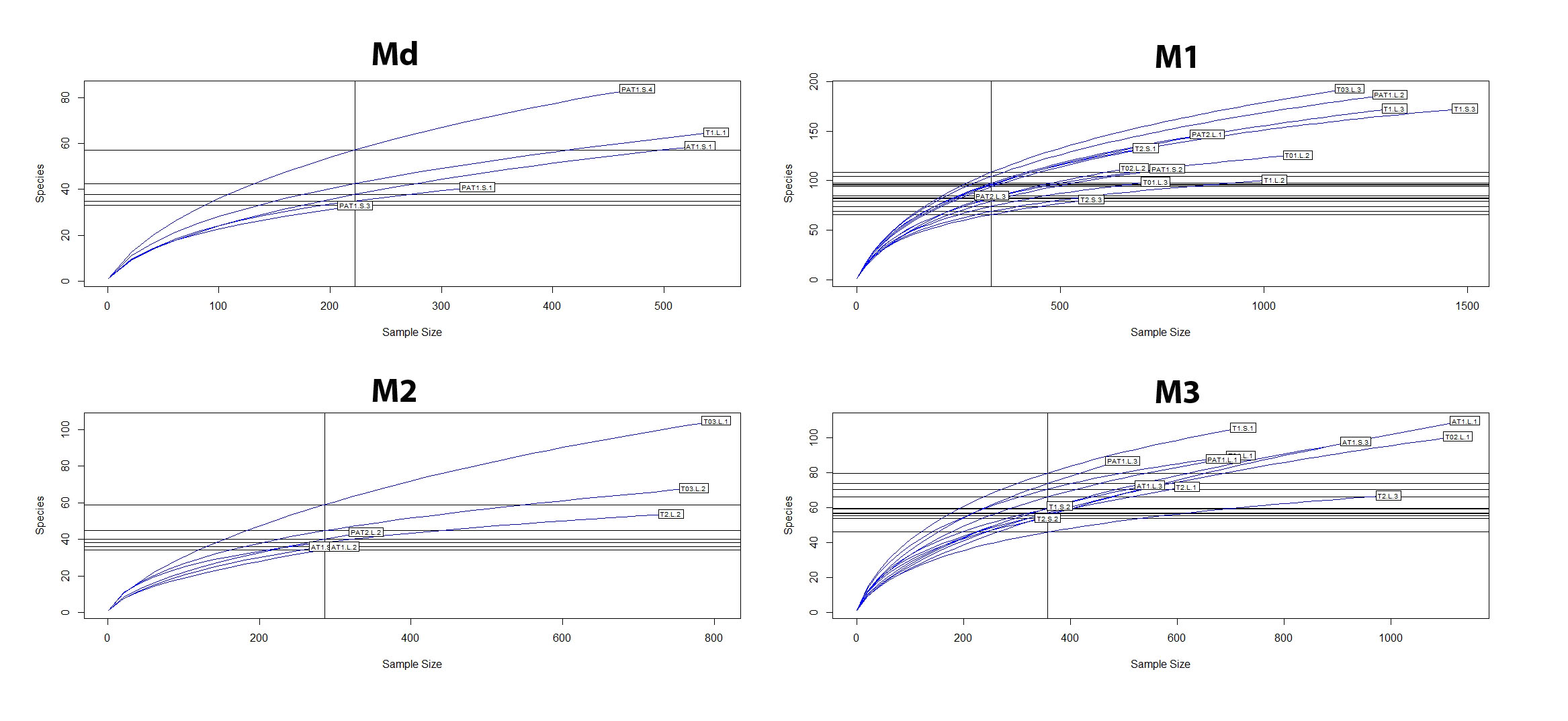
Additional file 3: Rarefaction curves of sequences from the different microbiotypes (Md, M1, M2 and M3).

Supplement: Supplementary file 4 — Additional file 3. Rarefaction curves of sequences from the different microbiotypes (Md, M1, M2 and M3). [file 40168_2020_835_MOESM3_ESM.docx]
