# Supplementary material for: Metabarcoding reveals distinct microbiotypes in the giant clam Tridacna maxima
Source: Microbiome. 2020 Apr 21;8:57. doi: 10.1186/s40168-020-00835-8 (PMC7175534; doi:10.1186/s40168-020-00835-8)
Supplement: Supplementary file 6 — Additional file 5. Principal Component Analysis build from PICRUSt2 analysis on the bacterial communities of T. maxima according to the microbiotypes. M: microbiotypes, Md: dying clam microbiotype; 1-3: clam microbiotype number. [file 40168_2020_835_MOESM5_ESM.pdf]

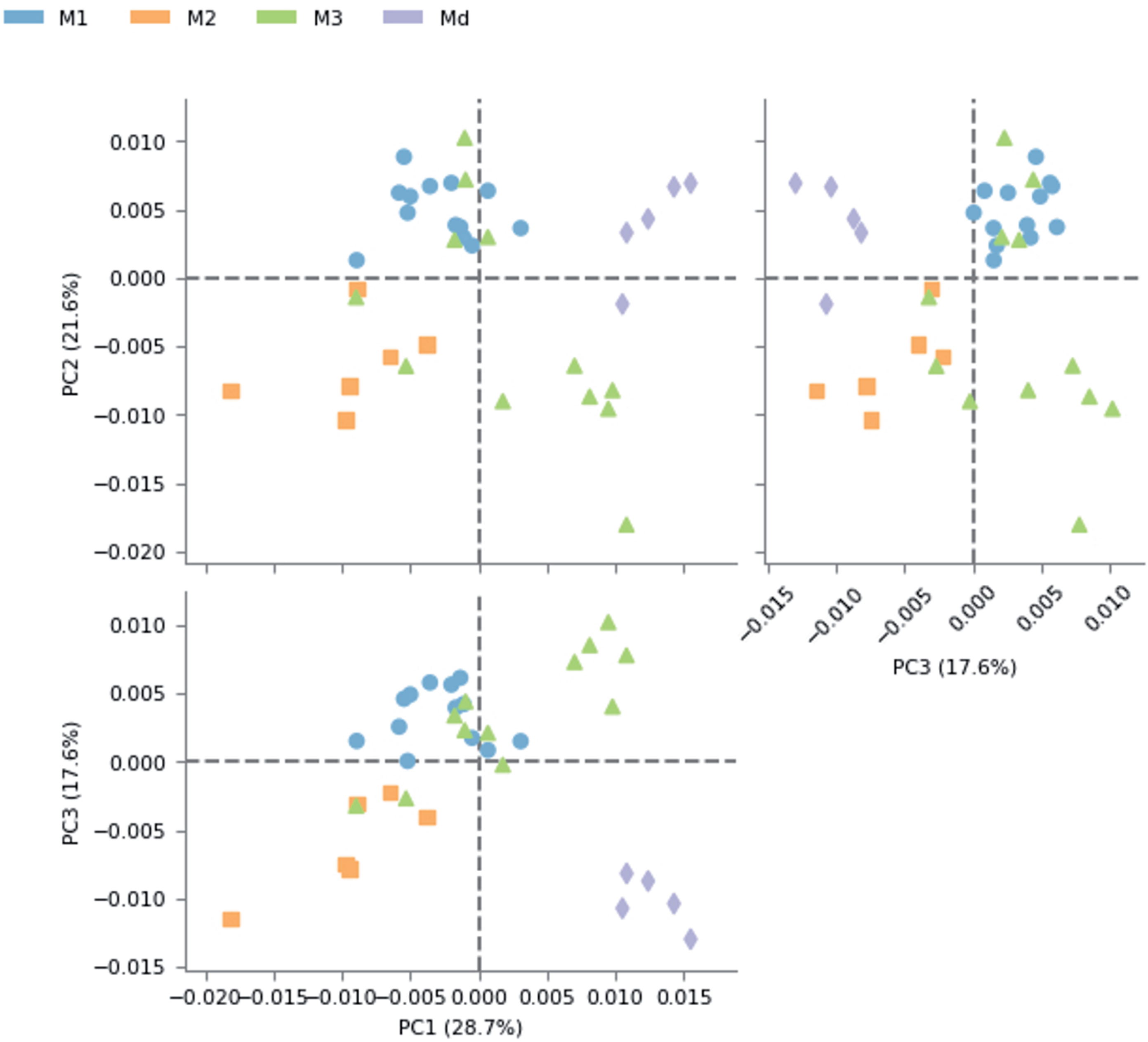

Additional file 5A: Principal Component Analysis build from PICRUSt2 analysis on the bacterial communities of T. maxima according to the microbiotypes. M: microbiotypes, Md: dying clam microbiotype; 1-3: clam microbiotype number.

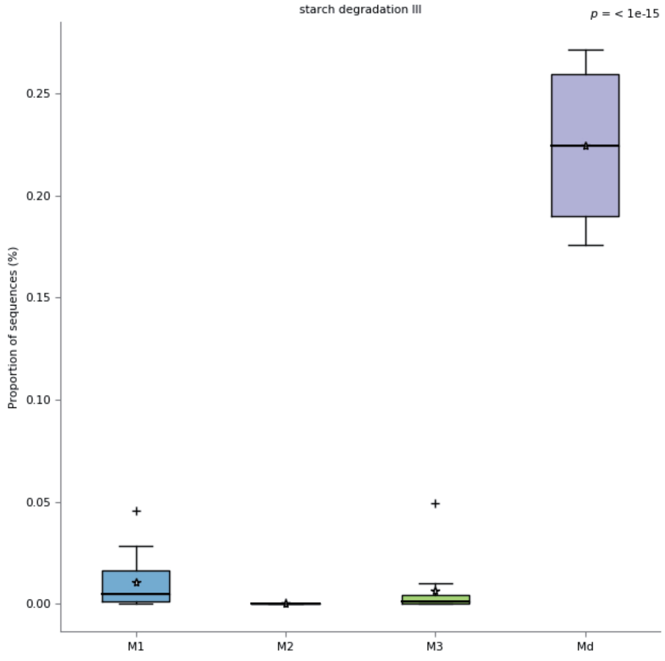
starch degradation III
p = < 1e-15
Proportion of sequences (%)
0.00
0.05
0.10
0.15
0.20
0.25
M1
M2
M3
Md

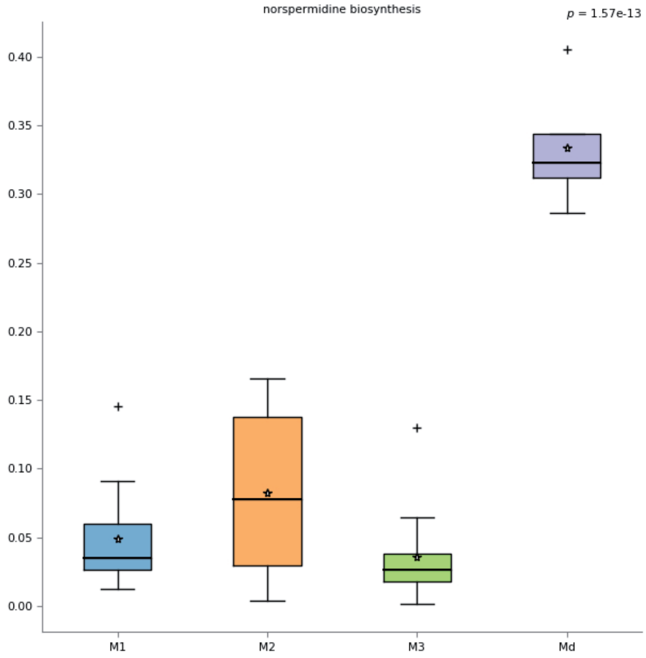
norspermidine biosynthesis
p = 1.57e-13
0.00
0.05
0.10
0.15
0.20
0.25
0.30
0.35
0.40
M1
M2
M3
Md

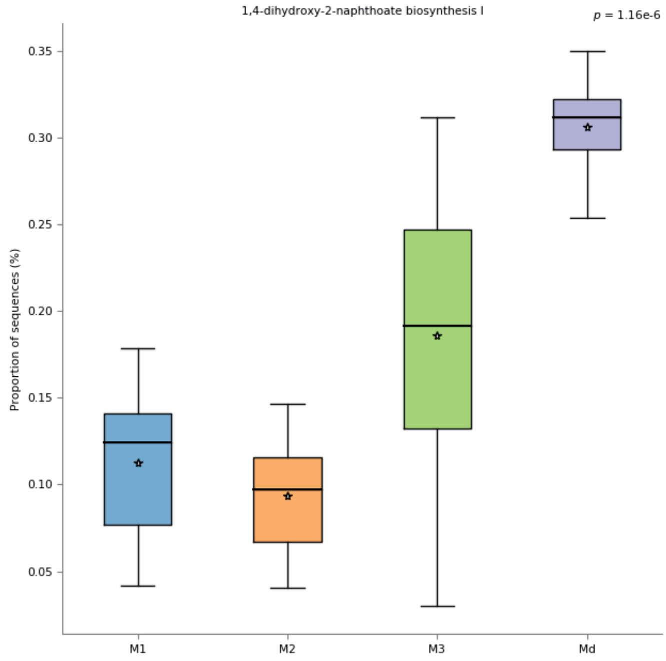
1,4-dihydroxy-2-naphthoate biosynthesis I
p = 1.16e-6
Proportion of sequences (%)
0.05
0.10
0.15
0.20
0.25
0.30
0.35
M1
M2
M3
Md

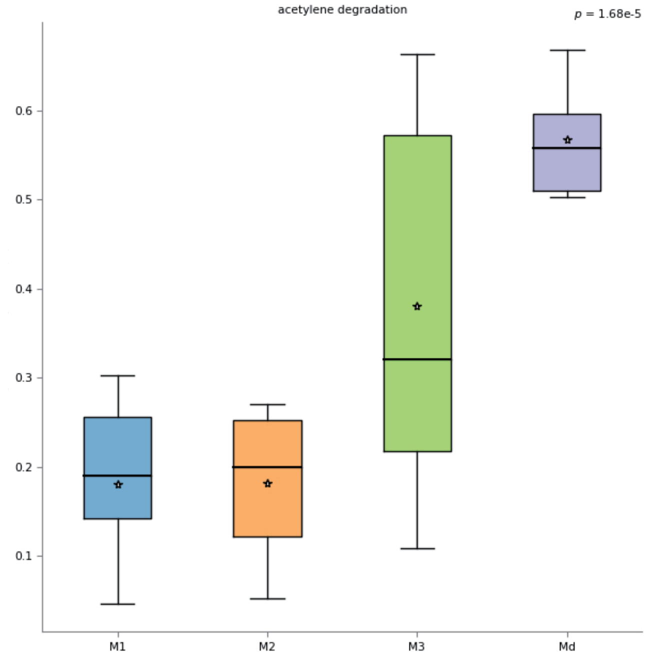
acetylene degradation
p = 1.68e-5
0.1
0.2
0.3
0.4
0.5
0.6
M1
M2
M3
Md

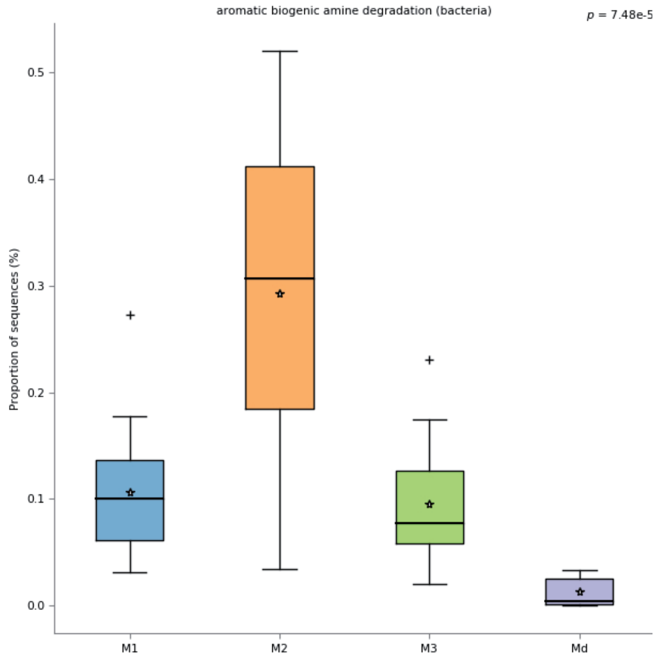
aromatic biogenic amine degradation (bacteria)
p = 7.48e-5
Proportion of sequences (%)
0.0
0.1
0.2
0.3
0.4
0.5
M1
M2
M3
Md

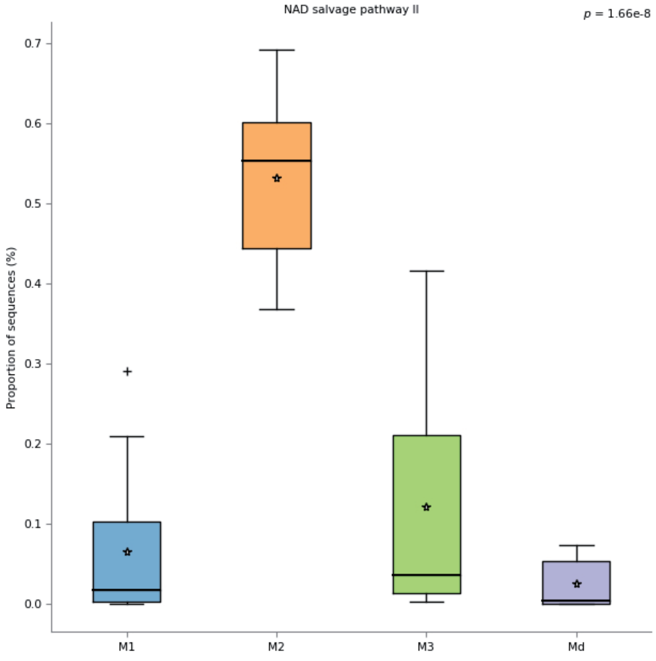
NAD salvage pathway II
p = 1.66e-8
Proportion of sequences (%)
0.0
0.1
0.2
0.3
0.4
0.5
0.6
0.7
M1
M2
M3
Md

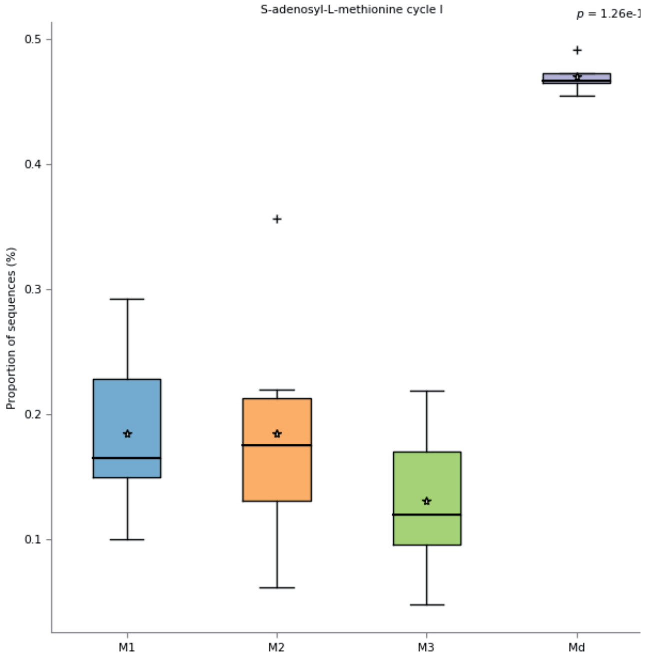
S-adenosyl-L-methionine cycle I
p = 1.26e-1
Proportion of sequences (%)
0.5
0.4
0.3
0.2
0.1
M1
M2
M3
Md

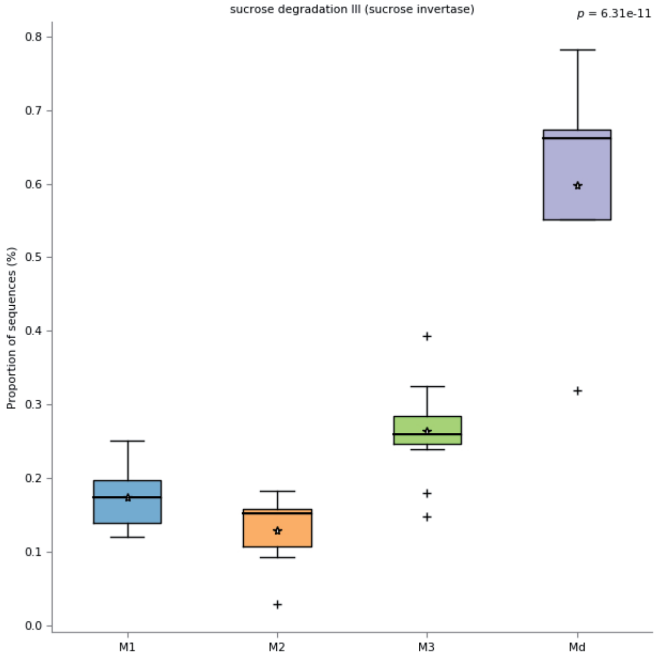
sucrose degradation III (sucrose invertase)
p = 6.31e-11
Proportion of sequences (%)
0.8
0.7
0.6
0.5
0.4
0.3
0.2
0.1
0.0
M1
M2
M3
Md

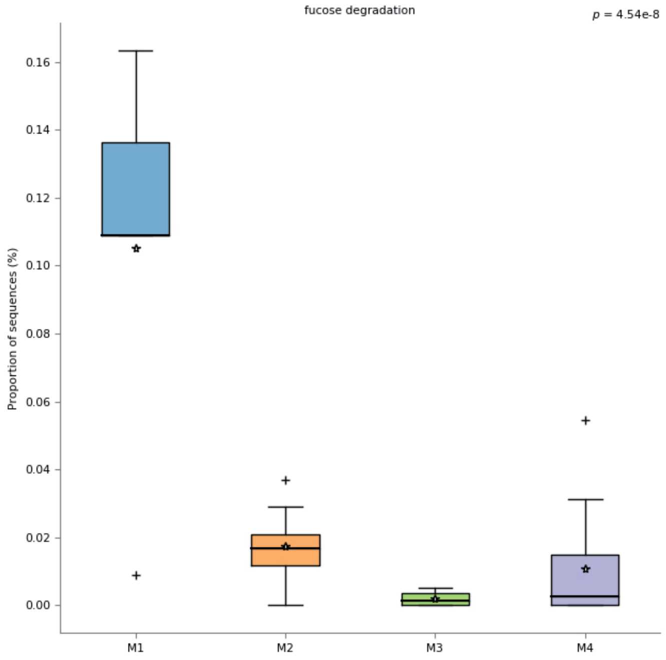
fucose degradation
p = 4.54e-8
Proportion of sequences (%)
0.16
0.14
0.12
0.10
0.08
0.06
0.04
0.02
0.00
M1
M2
M3
M4

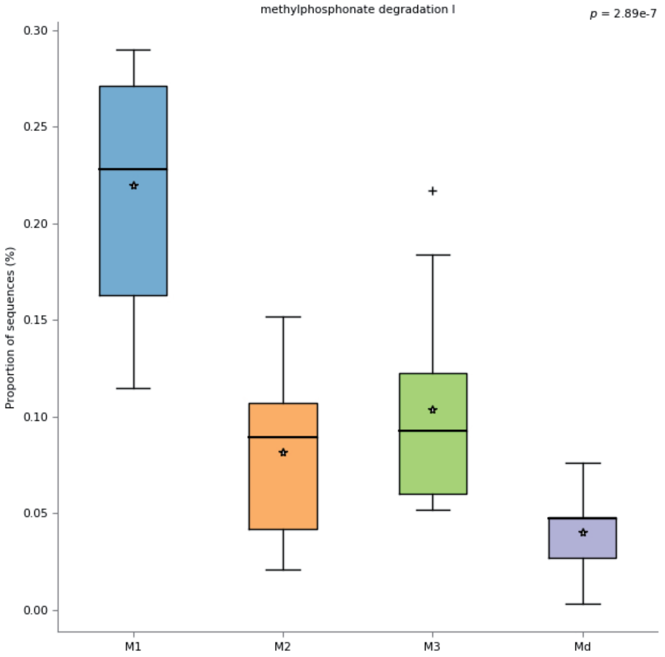
methylphosphonate degradation I
p = 2.89e-7
Proportion of sequences (%)
0.30
0.25
0.20
0.15
0.10
0.05
0.00
M1
M2
M3
Md

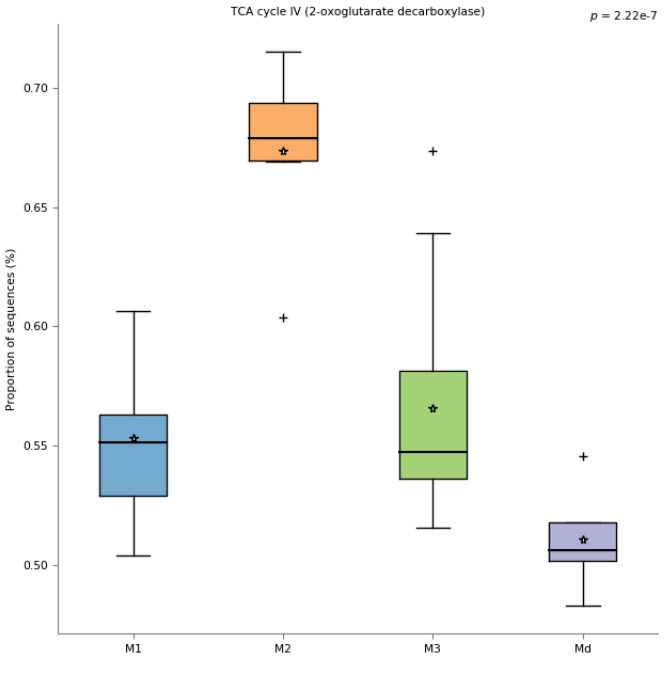
TCA cycle IV (2-oxoglutarate decarboxylase)
p = 2.22e-7
Proportion of sequences (%)
0.70
0.65
0.60
0.55
0.50
M1
M2
M3
Md

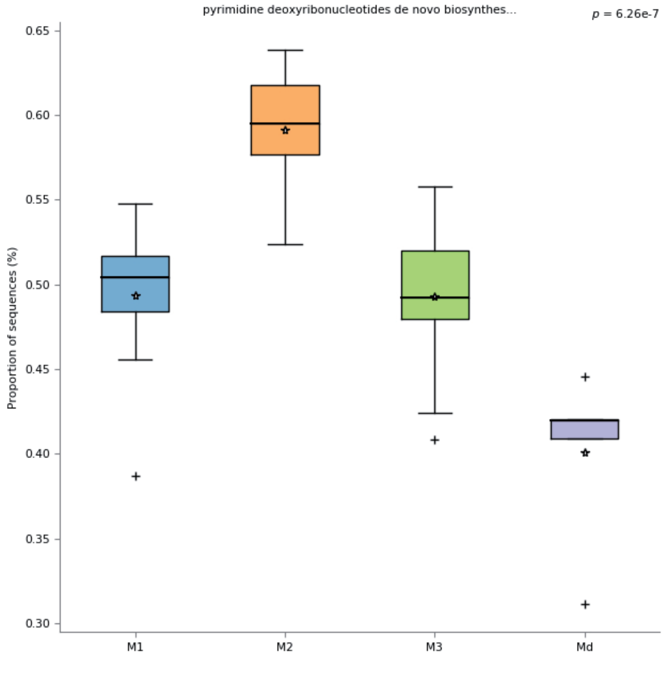
pyrimidine deoxyribonucleotides de novo biosynthes...
p = 6.26e-7
Proportion of sequences (%)
0.65
0.60
0.55
0.50
0.45
0.40
0.35
0.30
M1
M2
M3
Md

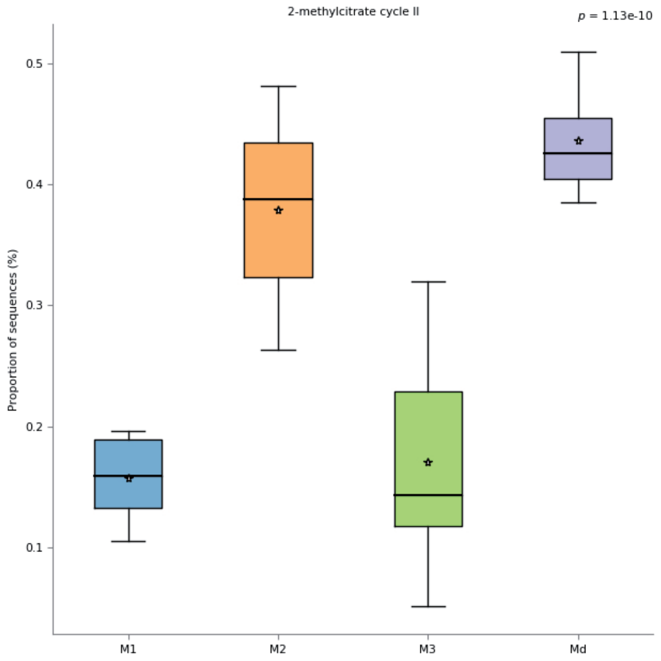

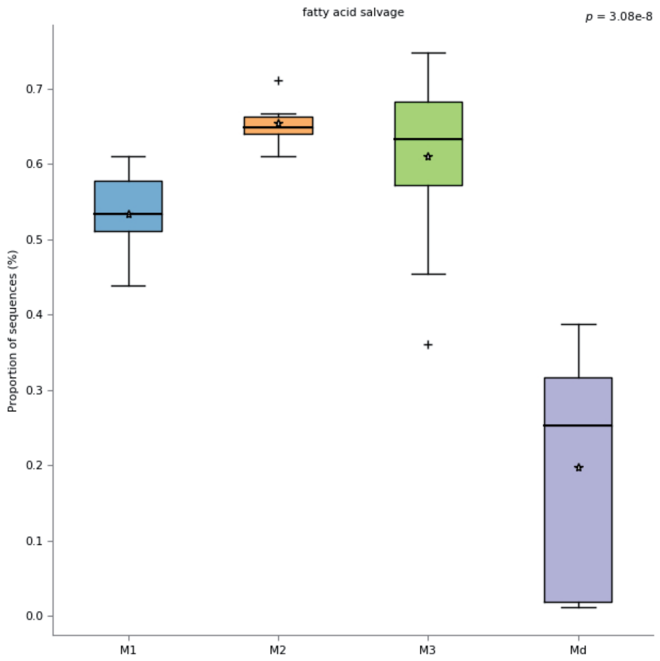

Additional file 5B: Boxplots of the microbiotype function analyses in Tridacna maxima performed with PICRUSt2.
