# Supplementary material for: Metabarcoding reveals distinct microbiotypes in the giant clam Tridacna maxima
Source: Microbiome. 2020 Apr 21;8:57. doi: 10.1186/s40168-020-00835-8 (PMC7175534; doi:10.1186/s40168-020-00835-8)
Supplement: Supplementary file 7 — Additional file 6. Relative abundance of Symbiodiniaceae subclades in Tridacna maxima determined by Illumina sequencing of the ITS2 DNA region (a) and the 23S rRNA gene (b). Abbreviations of experimental assemblages: PAT: P. damicornis, A. cytherea and T. maxima; AT: A. cytherea and T. maxima; T: T. maxima; 0: control; (1-3): experiment number; L: lagoon temperature; S: thermal stress; (1-4): sample number. [file 40168_2020_835_MOESM6_ESM.pdf]

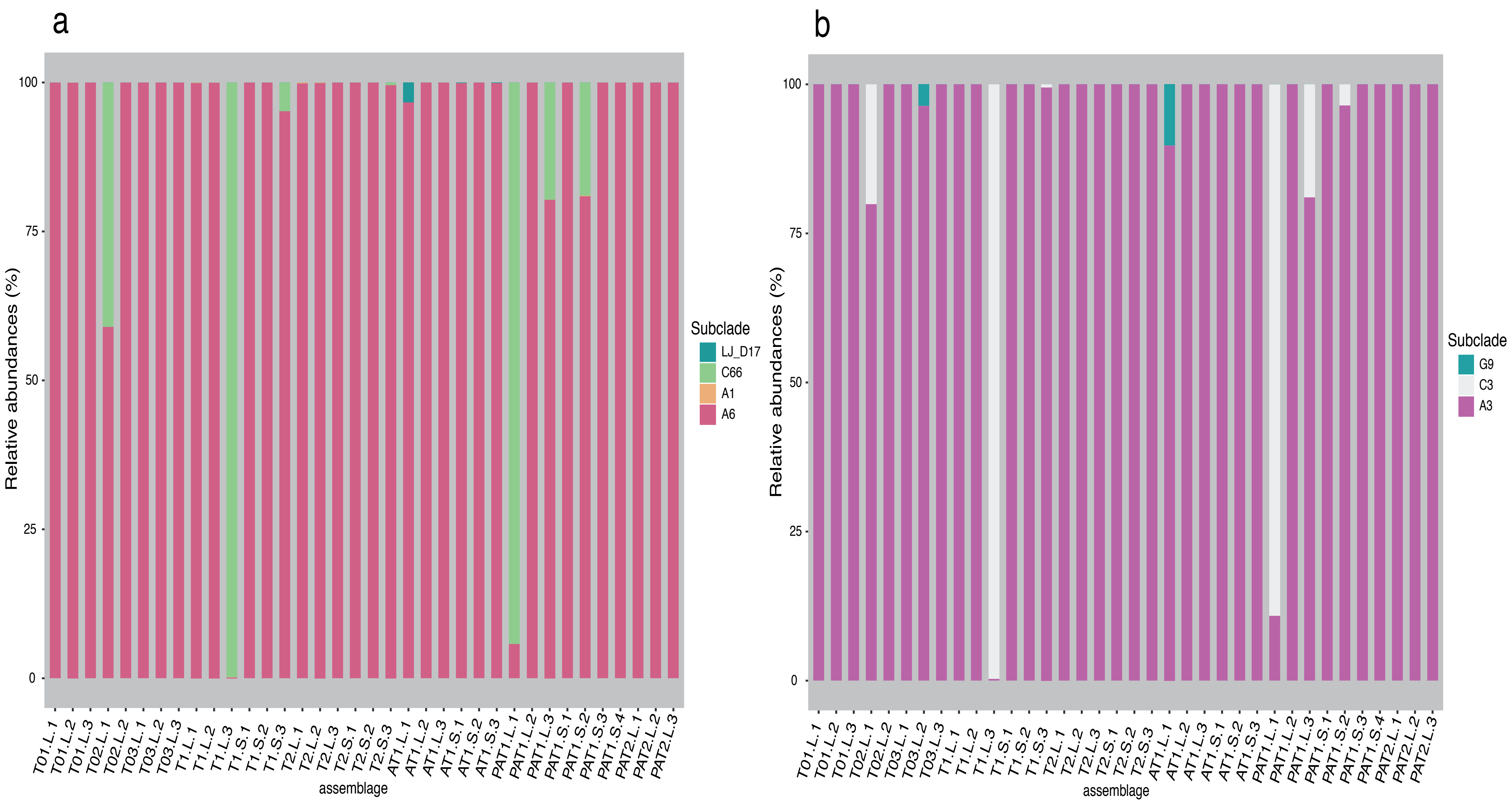

Additional File 8: Relative abundance of Symbiodiniaceae subclades in Tridacna maxima determined by illumine sequencing of the ITS2 DNA region (a) and the 23S rRNA gene (b). Abbreviations of experimental assemblages: PAT: P. damicornis, A. cytherea and T. maxima; AT: A. cytherea and T. maxima; T: T. maxima; 0: control; (1-3): experiment number; L: lagoon temperature; S: thermal stress; (1-4): sample number.
