# Supplementary material for: Metabarcoding reveals distinct microbiotypes in the giant clam Tridacna maxima
Source: Microbiome. 2020 Apr 21;8:57. doi: 10.1186/s40168-020-00835-8 (PMC7175534; doi:10.1186/s40168-020-00835-8)
Supplement: Supplementary file 8 — Additional file 7. Script of the bacterial community analysis performed with dada2. [file 40168_2020_835_MOESM7_ESM.docx]

Additional file 7: Script of the bacterial community analysis performed with dada2.

library("dada2")

path <- "./Bact"

list.files(path)

fnFs <- sort(list.files(path, pattern="_R1.fastq", full.names = TRUE))

fnRs <- sort(list.files(path, pattern="_R2.fastq", full.names = TRUE))

sample.names <- sapply(strsplit(basename(fnFs), "_"), `[`, 1)

# Place filtered files in filtered/ subdirectory

filtFs <- file.path(path, "filtered", paste0(sample.names, "_F_filt.fastq.gz"))

filtRs <- file.path(path, "filtered", paste0(sample.names, "_R_filt.fastq.gz"))

out <- filterAndTrim(fnFs, filtFs, fnRs, filtRs,

maxN=0, maxEE=c(1,1), truncQ=2, rm.phix=TRUE,

compress=TRUE, multithread=TRUE)

#Errors's rates

errF <- learnErrors(filtFs, nbases=1E10, multithread=TRUE)

plotErrors(errF, nominalQ=TRUE)

errR <- learnErrors(filtRs, nbases=1E10, multithread=TRUE)

plotErrors(errR, nominalQ=TRUE)

#Dereplication

derepFs <- derepFastq(filtFs, verbose=TRUE)

derepRs <- derepFastq(filtRs, verbose=TRUE)

# Name the derep-class objects by the sample names

names(derepFs) <- sample.names

names(derepRs) <- sample.names

#Inference

dadaFs <- dada(derepFs, err=errF, multithread=TRUE, pool = T)

dadaFs[[1]]

dadaRs <- dada(derepRs, err=errR, multithread=TRUE, pool = T)

dadaRs[[1]]

#Merging

mergers <- mergePairs(dadaFs, derepFs, dadaRs, derepRs, verbose=TRUE)

#Abund

seqtab <- makeSequenceTable(mergers)

dim(seqtab)

# Inspect distribution of sequence lengths

table(nchar(getSequences(seqtab)))

#si longueur trop disparates

seqtab <- seqtab[,nchar(colnames(seqtab)) %in% seq(298,317)]

#Chimera

seqtab.nochim <- removeBimeraDenovo(seqtab, method="consensus", multithread=TRUE, verbose=TRUE)

dim(seqtab.nochim)

sum(seqtab.nochim)/sum(seqtab)

#Recup fasta

write.table(colnames(seqtab.nochim),'nochim.tab',sep='\t',quote=F)

system('sed 1d nochim.tab > nochim.fas')

system('sed -e \'s/^\d*/>noc&/g\' nochim.fas > nochim.fasta')

system('sed -e \'s/\t/\n/g\' nochim.fasta > nochim.fas')

system('fasta_formatter -w 0 -i nochim.fas -o nochim.fasta')

#Otu table

Otu<-seqtab.nochim

colnames(Otu)<-paste("noc",as.character(1:ncol(Otu)),sep='')

write.table(t(Otu),'nochim.otu',sep='\t',quote=F)

##############################################

#PICRUSt2

conda activate picrust2

picrust2_pipeline.py -s nochim.fasta -i nochim.otu -o picrust2_BenBact -p 20

add_descriptions.py -i picrust2_BenBact/pathways_out/path_abun_unstrat.tsv.gz -m METACYC -o picrust2_BenBact/pathways_out/path_abun_unstrat_descrip.tsv.gz

#############################################

#Analysis

STAMP profile: path_abun_unstrat_descrip.tsv.gz / metadata: Microtype.tsv
